# Evaluation of antibodies for western blot analysis of frataxin protein isoforms

**DOI:** 10.1016/j.jim.2019.07.001

**Published:** 2019-07-04

**Authors:** Liwei Weng, Qingqing Wang, Sixiang Yu, Xiaolu Yang, David R. Lynch, Clementina Mesaros, Ian A. Blair

**Affiliations:** a Penn/CHOP Center of Excellence in Friedreich’s Ataxia, University of Pennsylvania, Philadelphia, PA 19104, USA; b Penn SRP Center and Center of Excellence in Environmental Toxicology, Department of Systems Pharmacology and Translational Therapeutics, Perelman School of Medicine, University of Pennsylvania, Philadelphia, PA 19104, USA; c Department of Cancer Biology, Perelman School of Medicine, University of Pennsylvania, Philadelphia, PA 19104, USA; d Department of Pediatrics and Neurology, Children’s Hospital of Philadelphia, Philadelphia, PA 19104, USA

**Keywords:** Gene therapy, Mature frataxin, Mitochondrial protein, Mitochondrial processing peptidase, Friedreich’s ataxia, Frataxin

## Abstract

Frataxin is the protein that is down-regulated in Friedreich ataxia (FRDA), an autosomal recessive genetic disease caused by an intronic GAA repeat expansion in intron-1 of the *FXN* gene. The GAA repeats result in epigenetic silencing of the *FXN* gene and reduced expression of the cytosolic full-length frataxin (1−210) protein. Full length frataxin translocates to the mitochondria, leading to formation of mature frataxin (81–210) formed by cleavage of the mitochondrial targeting sequence at K-80 of the full-length protein. There are currently no approved treatments for FRDA, although experimental approaches involving up-regulation or replacement of mature frataxin protein through numerous approaches are being tested. Many of the pre-clinical studies of these experimental approaches are conducted in mouse and monkey models as well as in human cell lines. Consequently, well-validated antibodies are required for use in western blot analysis to determine whether levels of various forms of frataxin have been increased. Here we examined the specificity of five commercially available anti-frataxin antibodies and determined whether they detect mature frataxin in mouse heart tissue. Four protein standards of monkey, human, and mouse frataxin as well as mouse heart tissue were examined using polyacrylamide gel electrophoresis (PAGE) in combination with western blot analysis. One antibody failed to detect any of the frataxin standards or endogenous frataxin in mouse heart tissue. Three of the antibodies detected a protein in mouse heart tissue that ran slightly faster on PAGE (at 23.4 kDa) to that predicted for full-length frataxin (23.9 kDa). One antibody detected all four frataxin standards as well as endogenous mouse mature frataxin in mouse tissue. Significantly, this antibody, which will be useful for monitoring mature frataxin levels in monkey, human, and mouse tissues, did not detect a protein in mouse heart tissue at 23.4 kDa. Therefore, antibodies detecting the immunoreactive protein at 23.4 kDa could be misleading when testing for the up-regulation of frataxin in animal models.

## 1. Introduction

Friedreich’s ataxia (FRDA) is an autosomal recessive disease caused primarily by an intronic GAA triplet expansion in the *FXN* gene, leading to reduced expression of full-length frataxin protein (Lynch et al., 2002) and the mitochondrial processing peptidase (MPP)-derived mature frataxin (Condo et al., 2007; Schmucker et al., 2008b). A small number of patients are heterozygous for GAA repeats in one allele and an *FXN* mutation in the other (< 4%); these individuals also have reduced levels of mitochondrial mature frataxin (Campuzano et al., 1996; Cossee et al., 1999; Delatycki et al., 2000; Santos et al., 2010). FRDA is estimated to affect 1 in 50,000 in the US population with an average age of death at 37 years, most commonly from cardiac-related pathologies (Pousset et al., 2015). The *FXN* gene encodes a mitochondrial protein frataxin, a highly conserved protein found in both prokaryotes and eukaryotes (Bencze et al., 2006; Pastore and Puccio, 2013). There is evidence to suggest that the GAA repeats induce epigenetic changes and heterochromatin formation, thereby impeding gene transcription (Al-Mahdawi et al., 2008). For example, studies conducted using blood from FRDA patients and lymphoblastoid cell lines have detected increased DNA methylation of specific CpG sites upstream of the GAA repeat and found histone modifications are present in regions flanking the GAA repeats (Greene et al., 2007). Thus, FRDA alleles become associated with histones that are hypoacetylated and show more extensive DNA methylation in the region flanking the repeat. It has been suggested that the hypoacetylation and extensive DNA methylation result in the formation of a compact chromatin configuration, with reduced binding of transcription factors so that frataxin transcription initiation and elongation are reduced (Kumari and Usdin, 2012). Furthermore, the number of GAA repeats correlate inversely with disease onset and directly with progression of the disease (Friedman et al., 2010; Metz et al., 2013; Patel et al., 2016). Although the exact role of frataxin has not been completely delineated, compelling evidence indicates that it is involved in the creation of iron-sulfur clusters. It could serve as an iron donor or regulate cysteine-derived persulfide formation during the assembly of mitochondrial iron-sulfur complexes (Lill et al., 2012; Fox et al., 2015; Braymer and Lill, 2017). The deficiency in frataxin protein leads to decreased ATP production by the iron-sulfur cluster (Bürk, 2017; Chiang et al., 2018).

**Fig. 1.**
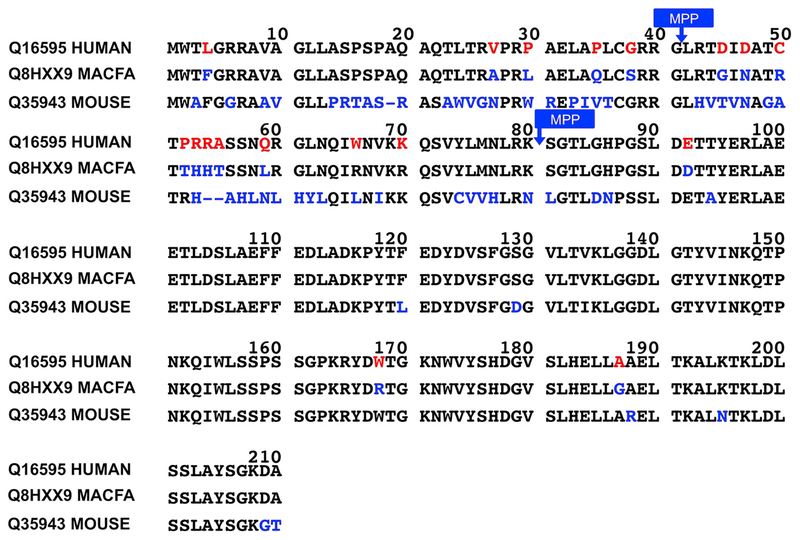
Comparison of the sequences of frataxin isoforms from three species. Sequences obtained from Uniprot.

The major *FXN* mRNA encodes the full-length 210-amino acid form of human frataxin with a molecular weight (MW) of 23,135 Da (Bencze et al., 2006). The corresponding cynomolgus monkey frataxin (1–210) and mouse frataxin (1–207) isoforms have MWs of 23,133 Da and 22,924 Da, respectively (Fig. 1). Upon being translated, the full-length human frataxin (1–210) is rapidly translocated from the cytosol to the mitochondria where a two-step process yields the mature biologically active form of human frataxin (Gakh et al., 2002a; Rufini et al., 2007; Schmucker et al., 2008a). MPP initially cleaves at the R-2 site between G41-L42 to give rise to an intermediate form of human frataxin (42–210), a 169-amino acid protein with a MW of 18,826 Da (Gakh et al., 2002b). The corresponding cynomolgus monkey intermediate frataxin (42–210) and mouse intermediate frataxin (41–207) isoforms have MWs of 18,741 Da and 18,574 Da, respectively (Fig. 1). The human intermediate frataxin then undergoes a second MPP-mediated cleavage at K80-S81 (a second R-2 site) to yield the mature active form of human frataxin (81–210), a 130-amino acid protein with a MW of 14,268 Da (Fig. 1) (Condo et al., 2007; Schmucker et al., 2008b). The corresponding cynomolgus monkey mature frataxin (81–210) and mouse mature frataxin (78–207) isoforms are also 130 amino acid proteins with MWs of 14,210 Da and 14,380 Da, respectively (Fig. 1). Additional minor human *FXN* alternative transcripts been reported (Campuzano et al., 1996; Pianese et al., 2002; Xia et al., 2012; Pérez-Luz et al., 2015), and we recently reported a novel frataxin protein of 135 amino acids (76–210, isoform E, MW 14,953 Da) found in human erythrocytes (Guo et al., 2018a).

There are currently no approved approaches for treating FRDA or slowing the progression of its symptoms (Strawser et al., 2017). Numerous therapeutic approaches are currently being tested, with the majority involving up-regulation or replacement of frataxin protein (Vyas et al., 2012; Wilson, 2012; Evans-Galea et al., 2014; Aranca et al., 2016; Kearney et al., 2016; Lynch et al., 2019). As an alternative to classical pharmacologically-based approaches, gene therapy and CRISPR-based methods are continuing to progress rapidly to become a promising potential therapeutic approach to increase frataxin expression (Evans-Galea et al., 2014; Li et al., 2015; Ouellet et al., 2017; Strawser et al., 2017). Most of the pre-clinical gene therapy and CRISPR approaches have been conducted in mouse models and/or human cells. We anticipate that monkey models will also be used for pre-clinical gene therapy studies before such treatments are tested on human FRDA patients. The adeno-associated virus 9 (AAV9) has been reported efficiently delivers virus to a conditional mouse model with severe cardiomyopathy caused by complete frataxin deletion in cardiac and skeletal muscle. The injection of AAV9-frataxin reverses the functional features of cardiomyopathy (Perdomini et al., 2014). In addition, AAV9-c to typical FRDA mouse models (Gérard et al., 2014). To circumvent the possible toxic effect caused by overexpression of frataxin, other approaches such as CRISPR/Casp9 were also tested on mouse models (Li et al., 2015; Ouellet et al., 2017). The expression levels of full length frataxin protein in the gene therapy studies are usually monitored by quantifying the corresponding mRNA levels. However, this will not necessarily reflect the amount of mitochondrial mature frataxin that is expressed. Therefore, specific and sensitive methodology is required in order to reliably analyze expression of mature frataxin in tissue samples. Availability of this methodology would also enable surrogate tissues to be identified so that mature frataxin expression can be monitored in human subjects. In the present study, we report a screening of five antibodies in order to identify those that would be useful for conducting such studies using PAGE/western blot of tissue samples.

## 2. Materials and methods

### 2.1. Materials

Four protein standards were used in this study. Cynomolgus monkey mature frataxin (aa 81–210) was purchased from LifeSpan BioSciences (LS-G21788). Mouse recombinant intermediate form of frataxin (aa 41–207) was obtained from LifeSpan BioSciences (LS-G14956). Human and mouse mature frataxin were prepared using a protocol described previously (Guo et al. Anal. Chem.). Five antibodies were tested here, including AB113691, AB175402, AB124680 obtained from Abcam (Cambridge, MA), MAB1594 from Millipore-Sigma (Billerica, MA), and LS-C197243 from LifeSpan BioSciences (Seattle, WA). Anti-rabbit HRP and anti-mouse HRP were obtained from Santa Cruz Biotechnology, Inc. (Dallas, TX). Stainless steel beads for tissue homogenization were purchased from Next Advance (Troy, NY). NuPAGE™ LDS sample buffer was from Thermo Scientific (Waltham, MA) and chemiluminescence reagent (NEL103E001) was purchased from Perkin Elmer (Waltham, WA). Costar® Spin-X centrifuge tube filters (0.22 μm cellulose acetate) were obtained from Corning (Corning, NY).

### 2.2. Tissue homogenate

The heart samples were from WT C57BL/6 mouse, and were provided by Dr. Wellen (Associate Professor of Cancer Biology, Perelman School of Medicine, University of Pennsylvania). All surgery experiments were performed in accordance with protocols approved by the Institutional Animal Care and Use Committee (IACUC) of the University of Pennsylvania. A piece of heart tissue sample was weighed (20–100 mg) and cut into 2–3 mm^2^ pieces. All the pieces were collected in an Eppendorf tube and immunoprecipitation (IP) lysis buffer [150 mM sodium chloride, 50 mM tris(hydroxymethyl)aminomethane (Tris). HCl pH 7.5, 1 mM ethylenediaminetetraacetic acid (EDTA), 0.5% Triton X-100, 0.5% NP-40, 1 mM dithiothreitol (DTT)] with 0.5% SDS was added to make the concentration of 100 mg/mL. Approximately, 30–50 stainless steel beads (0.9–2.0 mm) were added to the mixture. The homogenization was carried out twice using Bullet Blender® Gold (Next Advance Inc., Troy, NY) at a speed of 6 for 4 min. If white fibers were still present in the samples, they were further homogenized using probe sonication at strength of 5 for 30 pulses. Following centrifugation at 17720×*g* for 5 min, a portion of the supernatant was withdrawn to mix with 1 × NuPAGE™ LDS sample buffer containing 2% β-mercaptoethanol and boiled at 95 °C for 5 min. After being briefly cooled down, the samples were filtered through Costar® Spin-X centrifuge tube filter (0.22 μm cellulose acetate) before being loaded on 12% SDS PAGE.

### 2.3. Western blotting

A portion of tissue homogenate or protein standards including the intermediate form of mouse frataxin and the mature form of monkey frataxin, human frataxin, and mouse frataxin were mixed with 1×NuPAGE™ LDS sample buffer containing 2% β-mercaptoethanol. The mixtures were boiled 95 °C for 5 min and cooled down to room temperature, the supernatants were loaded on 12% SDS PAGE. The gel was run at 100 V for about 80 min until the dye was at the bottom. Typically, 200–400 μg of tissue was analyzed for each western blot. There was 0.03–0.07 mg protein/mg tissue and so 6–28 μg of total protein was load in each lane. Intense signals were observed for the protein standards showing that the proteins were transferred well to the membrane. This was confirmed by Ponceau staining after transferring to the membrane, which showed that of all the high abundant protein bands in the tissue samples were present. Proteins were transferred to an Invitrogen nitrocellulose membrane with a pore size of 0.2 μm (Thermo Scientific, Waltham, MA). Membranes were blocked for 1 h at room temperature with 5% milk in Dulbecco’s phosphate-buffered saline containing 0.1% Tween-20 (DPBST), then incubated with a range of anti-Frataxin antibodies that were diluted using 5% non-fat dry milk in DPBST as instructed by the manufacture at 4 °C for overnight. Ab113691 and Ab175402 were diluted 1:500, and Ab124680, MAB1594 and LS-C197243 were diluted 1:1000. The membranes were washed in DPBST for 15 min 3 times before being incubated with the secondary Ab, either anti-rabbit HRP 1:5000 or anti-mouse HRP 1:5000, as appropriate. Chemiluminescence was generated using NEL103E001 and captured by X-ray films that were developed using a Konica Minolta SRX-101A medical film processor (Browns Medical Imaging, Omaha, NE). The amount of sample loaded on the gel was adjusted so that bands could be observed with a similar intensity after a similar exposure time (30–45 s). Each western blot was performed at 3–5 times with very consistent results. Western blots using different antibodies were performed separately. The standards that were obtained commercially had defined concentrations so they served as loading controls. Control experiments were conducted without the primary antibody to show that the detected bands were specific to that antibody.

## 3. Results

### 3.1. Analysis of human and mouse mature frataxin

**Fig. 2.**
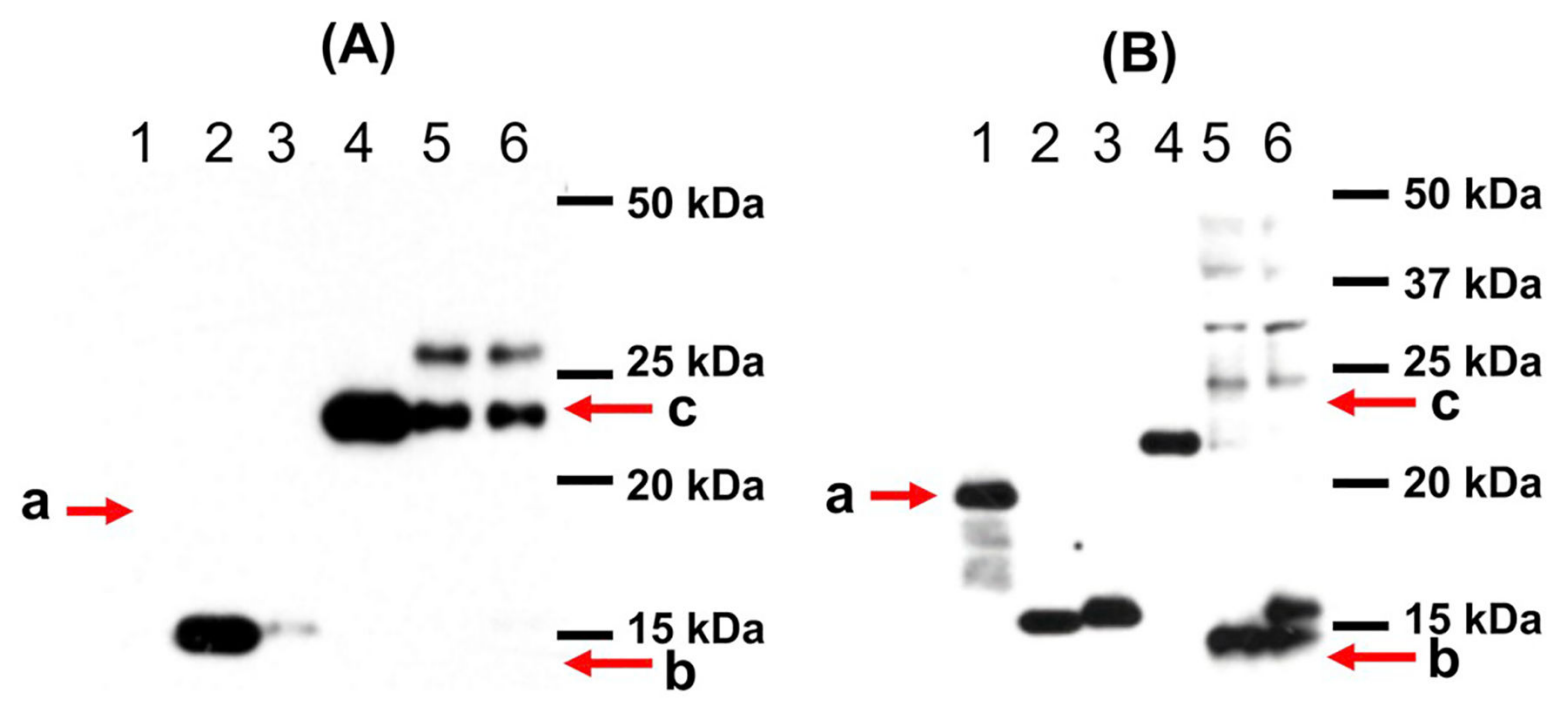
Western blot images of frataxin standards and mouse tissue samples. (A) Abcam Ab113691. (B) Abcam Ab175402. Lane 1: Monkey mature His-tag-frataxin standard (a, 200 pg; 18.3 kDa); lane 2: Human mature His-tag-frataxin standard (200 pg; 15.3 kDa); lane 3: Mouse mature His-tag-frataxin standard (200 pg; 15.4 kDa); lane 4: Mouse intermediate His-tag-frataxin standard (200 pg, 22.4 kDa); lane 5: Mouse heart homogenate (400 μg); lane 6: Mouse heart homogenate (400 μg) + mouse mature His-tag-frataxin standard (200 pg). b: Expected mobility for mouse mature frataxin (14.3 kDa). c: Expected mobility for mouse full-length frataxin (22.9 kDa).

**Fig. 3.**
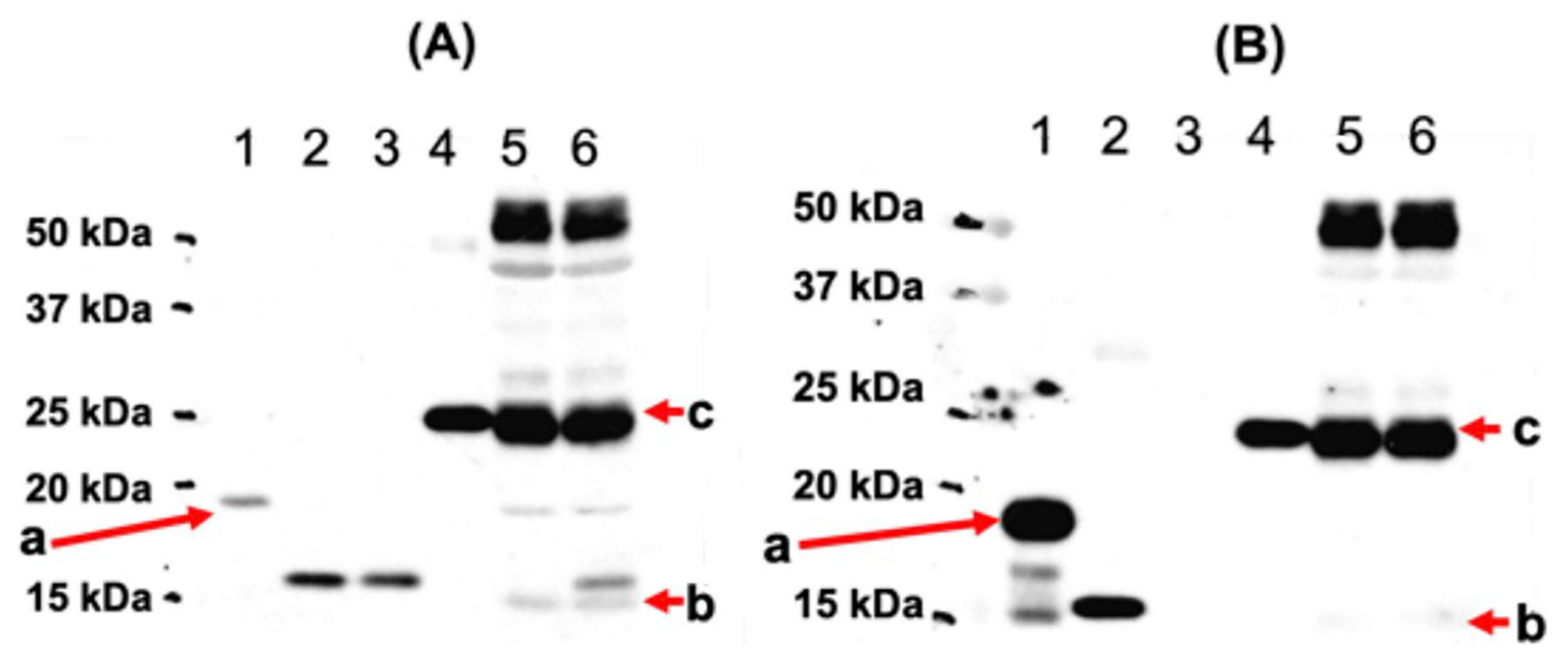
Western blot image of frataxin standards and mouse tissue samples. (A) Millipore Sigma MAB1594. (B) LifeSpan Biosciences LS-C197243. Lane 1: Monkey mature His-tag-frataxin standard (a; 500 pg; 18.3 kDa); lane 2: Human mature His-tag-frataxin standard (500 pg; 15.3 kDa); lane 3: Mouse mature His-tag-frataxin standard (500 pg; 15.4 kDa); lane 4: Mouse intermediate His-tag-frataxin standard (500 pg, 22.4 kDa); lane 5: Mouse heart homogenate (200 μg); lane 6: Mouse heart homogenate (200 μg) + mouse mature His-tag-frataxin standard (500 pg). b: Expected mobility for mouse mature frataxin (14.3 kDa). c: Expected mobility for mouse full-length frataxin (22.9 kDa).

**Fig. 4.**
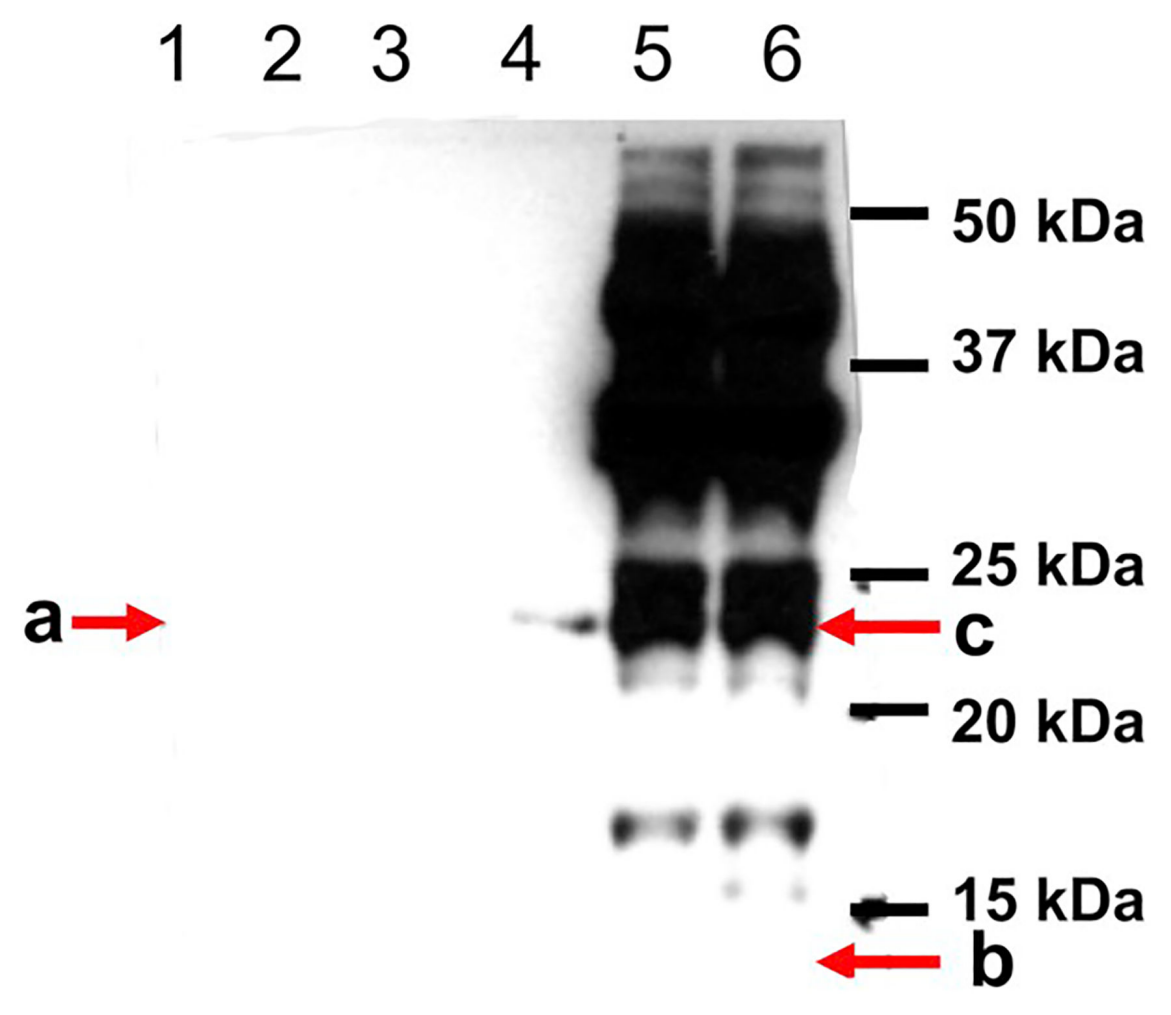
Western blot images of frataxin standards and mouse tissue samples blotted with Abcam Ab124680. Lane 1: Monkey mature His-tag-frataxin standard (a; 300 pg; 18.3 kDa); lane 2: Human mature His-tag-frataxin standard (300 pg; 15.4 kDa); lane 3: Mouse mature His-tag-frataxin standard (500 pg; 15.3 kDa); lane 4: Mouse intermediate His-tag-frataxin standard (300 pg, 22.4 kDa); lane 5: Mouse heart homogenate (300 μg); lane 6: Mouse heart homogenate (300 μg) + mouse mature His-tag-frataxin standard (500 pg). b: Expected mobility for mouse mature frataxin (14.3 kDa). c: Expected mobility for mouse full-length frataxin (22.9 kDa).

Human and mouse mature frataxin, which were prepared as described with His-tags at their C-termini (for purification) (Guo et al., 2018b), had MWs of 15.3 Da and 15.4 kDa, respectively. The protein standards were subjected to SDS-PAGE followed by western blotting with all five selected antibodies. Four out of five tested antibodies detected human mature frataxin, but with different sensitivities (Figs. 2–4). The two Abs purchased from Abcam (AB113691 and AB175402) and the mAb from LifeSpan Bio (LS-C197243) had the highest sensitivity for human frataxin (Figs. 2A, B, and 3B; Table 2). Both of the Abcam Abs were prepared using recombinant full-length human frataxin as the immunogen (Table 1). The LifeSpan Bio mAb was raised against a peptide from human mature frataxin (aa 91–200) with GST tag (Table 1, Fig. 3B). Human mature frataxin showed low intensity when blotted with the mAb from Millipore-Sigma (MAB1594), even though a larger amount of the protein was loaded on the gel (Fig. 3A). Interestingly, this mAb was also raised against full length human frataxin, although fused to TrpE. The Ab (AB124680) prepared using a synthetic peptide corresponding to human frataxin aa 150 to the C-terminus as the immunogen (the full amino acid sequence was not divulged from the manufacture due to protection of intellectual property) did not recognize human mature frataxin at all (Fig. 4). Of the three Abs that were supposed to recognize mouse frataxin, AB175402 showed the best immunoreactivity (Fig. 2B), MAB1594 was less sensitive (Fig. 3A), and AB113691 barely recognized the mouse mature frataxin standard (Fig. 2A). The other two mAbs (AB124680 and LS-C197243) could not detect mouse mature frataxin standard, as expected from the supplier’s description (Figs. 3B and 4).

### 3.2. Analysis of cynomolgus monkey mature frataxin

Cynomolgus monkey mature frataxin was expressed in *E. coli*, with a 4 kDa tag at the N-terminus and so the migration for this protein corresponded to 18.3 kDa. Two of the Abs that were tested (AB175402 and LS-C197243) recognized the monkey mature frataxin standard with good intensity (Figs. 2B and 3B). MAB1594 weakly detected it when a larger amount was loaded on the gel (Fig. 3A). AB113691, which detected human mature frataxin with good intensity, did not recognize mature monkey frataxin at all (Fig. 2A). This was very surprising in view of the sequence similarity between human and monkey frataxin (Fig. 1).

### 3.3. Analysis of mature frataxin in mouse heart tissue

Mouse heart tissue was homogenized and filtered before being analyzed by SDS-PAGE. A mouse intermediate frataxin form purchased from Lifespan BioSciences was also loaded on the side as a control. The intermediate standard was expressed with a 4 kDa His-tag according to the manufacture and so migrated at 22.4 kDa. Surprisingly, AB113691, which barely detected mouse mature frataxin, recognized the mouse intermediate standard with high sensitivity (Fig. 2A). It also detected two proteins in the mouse tissue samples, with one protein migrating the same as the mouse intermediate frataxin standard (22.4 kDa), the other migrating slower (27 kDa). Endogenous intermediate frataxin (41–207) is an 18.5 kDa protein whereas full-length mouse frataxin has a MW of 22.9 kDa. Therefore, the two proteins are most likely immunoreactive proteins detected by the Ab that are un-related to frataxin. This mAb also failed to detect either endogenous mouse mature frataxin or the mouse mature frataxin standard that was added to the tissue samples (Fig. 2A). AB175402 recognized both the endogenous mature mouse frataxin and the mouse mature frataxin standard that was spiked into the tissue samples (Fig. 2B). It also recognized the mouse intermediate standard but did not recognize either of immunoreactive proteins in the heart tissue at 22.4 and 27 kDa (Fig. 2B). Ab124680 detected numerous immunoreactive proteins in the mouse heart tissue but it was not clear whether any of them corresponded to a frataxin isoform (Fig. 4). Conversely, Ab124680 showed weak immunoreaction to mouse intermediate standard and no band was founded corresponding to endogenous mouse mature form (Fig. 4). MAB1594 weakly recognized endogenous mature frataxin in the mouse heart tissue and also weakly recognized the mouse mature frataxin standard that was spiked into the tissue (Fig. 3A). In contrast, MAB1594 recognized the mouse intermediate standard with high sensitivity and the protein that migrated with the same mobility as the mouse intermediate frataxin standard at 22.4 kDa as well as some higher MW proteins (Fig. 4A). LS-C197243 did not recognize endogenous mature frataxin in the mouse heart tissue or the mouse mature frataxin standard that was spiked into the tissue (Fig. 3B). However, it did recognize the mouse intermediate standard with high sensitivity and the protein that migrated with the same mobility as the mouse intermediate frataxin standard at 22.4 kDa as well as some higher MW proteins (Fig. 3B).

## 4. Discussion and conclusion

Human, monkey and mouse frataxin sequences were down-loaded from Uniprot and aligned with the relevant Uniprot program. Full-length frataxin (1–210) from human and monkey frataxin share 91% sequence similarity while human full-length frataxin (1–210) and mouse full-length frataxin (1–207) share over 72% sequence similarity (Fig. 1). The sequence of intermediate frataxin (42–210) from human and monkey are over 92% similar whereas human intermediate frataxin (42–210) and mouse intermediate frataxin (41–207) share 78% sequence similarity. Human and monkey mature frataxin (81–210) differ by only 3 amino acids (E92D, W168R, A186G), while the human mature frataxin (81–210) and mouse mature frataxin (78–207) differ by 11 amino acids (Fig. 1). Of the five Abs that were tested, the suppliers indicated that five of them were immunoreactive with human frataxin; whereas, three suppliers indicated that their Abs were immunoreactive with both human and mouse frataxin (AB113691, AB175402 and MAB1594). Due to the high sequence similarity between monkey and human frataxin, it was highly likely that the selected Abs would also recognize monkey frataxin, although none of the suppliers claimed that they would.

Clearly, the mAb obtained from LifeSpan Bio (LS-C197243) was the best for differentiating human and mouse mature frataxin (Fig. 3B). It recognized human mature frataxin but did not recognize endogenous mature frataxin in the mouse heart tissue or the mouse mature frataxin standard that was spiked into the tissue (Fig. 3B). Therefore, this mAb will be useful in mouse models such as the siRNA knockdown mouse (Chandran et al., 2017) to show that when treated with a human transgene, human frataxin protein is expressed. Curiously, this mAb detected a His-tagged mouse intermediate frataxin standard but not endogenous mouse mature frataxin (Fig. 3B). This suggests that the mAb could also be useful for confirming whether there is aberrant processing of the full-length mouse protein during pre-clinical gene therapy studies in mouse models.

Abcam Ab175402, was undoubtedly the best for detecting both human and mouse frataxin (Fig. 2B). It detected both endogenous mouse mature frataxin in mouse heart tissue as well as the mature frataxin standard spiked into the tissue samples. There was no loss in sensitivity as a result of Ab binding to tissue components when compared with the standard alone (Fig. 2B). This contrast with MAB1594 where there was a significant loss of intensity in the presence of the tissue sample as well as a reduction in intensity of the signal for endogenous frataxin (Fig. 3A) when compared with Ab175402 (Fig. 2B). Four of the Abs bound strongly to the mouse intermediate frataxin (41–207) standard (Table 2). This means that they would be useful for determining whether there is aberrant processing of full-length frataxin in the animal models. AB113691 was the only Ab tested that could differentiate cynomolgus monkey mature frataxin form from human mature frataxin (Fig. 2A). However, it is not clear if the large tag that was present at the amino terminus could have caused this loss of immunoreactivity. Additional experiments with cynomolgus monkey tissue coupled with expression of monkey frataxin with a shorter His-tag will be required to clarify this issue. AB175402, MAB1594, and LS-C197243 were able to detect both human and monkey frataxin without clear preference (Figs. 2B, 3A, and B). This means that if frataxin is up-regulated in a monkey model after gene therapy, it will be necessary to confirm the presence of the human frataxin isoform using mass spectrometry-based procedures such as those we have described recently (Guo et al., 2018a; Guo et al., 2018b). Finally, AB124680 was unable to detect any of the frataxin standards or endogenous frataxin in mouse heart tissue because of the high level of non-specific binding (Fig. 4).

In conclusion, an Ab has been identified that can distinguish human from mouse frataxin (LS-C197243), an Ab that binds to human, mouse and monkey frataxin (Ab175402), and an Ab that binds to human and mouse mature frataxin but not monkey mature frataxin (Ab113691). We anticipate that these Abs will be useful for analyzing frataxin expression in both pre-clinical animal and human cell models to determine whether human frataxin is up-regulated by novel therapeutic interventions such as gene therapy and CRISPR designed to correct the defect in frataxin production in Friedreich’s ataxia.

## Figures and Tables

**Table 1:** Information on the antibodies tested.

Supplier	Catalog number	Species	Clone	Monoclonal/polyclonal	Epitope reported
Abcam	Ab113691	Mouse	17A11	Monoclonal	Full length protein
Abcam	Ab175402	Rabbit	N/A	Polyclonal	Full length protein
Millipore-Sigma	MAB1594	Mouse	1G2	Monoclonal	Full length fused to TrpE
LifeSpan Bio	LS-C197243	Mouse	1D9	Monoclonal	Synthetic peptide (aa91–200) with GST tag
Abcam	Ab124680	Rabbit	EPR6107	Monoclonal	Synthetic peptide (aa 150 - C-terminus)

**Table 2:** Antibody characteristics.

Figure	Catalog number	His-tag monkey frataxin	His-tag human frataxin	His-tag mouse mature frataxin	His-tag mouse intermediate frataxin	Mouse Tissue		
						Endog mature frataxin	Immuno-reactive protein (22.4 kDa)	His-tag mouse frataxin
2A	Ab113691	None	Strong	Weak	Strong	None	Strong	None
2B	Ab175402	Strong	Strong	Strong	Strong	Strong	None	Strong
3A	MAB1594	Weak	Weak	Weak	Strong	Weak	Strong	Weak
3B	LS-C197243	Strong	Strong	None	Strong	None	Strong	None
4	Ab124680	None	None	None	Very weak	None	Interference	None

## Abbreviations:

Ab: Antibody
mAb: Monoclonal antibody
DTT: Dithiothreitol
DPBST: Dulbecco’s phosphate-buffered saline containing 0.1% Tween-20
EDTA: Ethylenediaminetetraacetic acid
FRDA: Friedreich’s ataxia
IP: Immunoprecipitation
MPP: Mitochondrial processing peptidase
MW: Molecular weight
PAGE: Polyacrylamide gel electrophoresis
SDS: Sodium dodecyl sulfate
Tris: Tris(hydroxymethyl)aminomethane
